# Probing eukaryotic cell mechanics via mesoscopic simulations

**DOI:** 10.1371/journal.pcbi.1005726

**Published:** 2017-09-18

**Authors:** Kirill Lykov, Yasaman Nematbakhsh, Menglin Shang, Chwee Teck Lim, Igor V. Pivkin

**Affiliations:** 1 Institute of Computational Science, Faculty of Informatics, USI Lugano, Lugano, Switzerland; 2 NUS Graduate School for Integrative Sciences and Engineering, National University of Singapore, Singapore, Singapore; 3 Department of Biomedical Engineering, National University of Singapore, Singapore, Singapore; 4 BioSystems and Micromechanics (BioSyM) IRG, Singapore-MIT Alliance for Research and Technology (SMART) Centre, Singapore, Singapore; 5 Mechanobiology Institute, National University of Singapore, Singapore, Singapore; 6 Swiss Institute of Bioinformatics, Lausanne, Switzerland; University of California Irvine, UNITED STATES

## Abstract

Cell mechanics has proven to be important in many biological processes. Although there is a number of experimental techniques which allow us to study mechanical properties of cell, there is still a lack of understanding of the role each sub-cellular component plays during cell deformations. We present a new mesoscopic particle-based eukaryotic cell model which explicitly describes cell membrane, nucleus and cytoskeleton. We employ Dissipative Particle Dynamics (DPD) method that provides us with the unified framework for modeling of a cell and its interactions in the flow. Data from micropipette aspiration experiments were used to define model parameters. The model was validated using data from microfluidic experiments. The validated model was then applied to study the impact of the sub-cellular components on the cell viscoelastic response in micropipette aspiration and microfluidic experiments.

## Author summary

Predictive simulations of cell flow in microfluidic devices and capillary networks may help to quantify the impact of different cell components on its behavior. Cells have complex mechanical properties and can undergo significant deformations, requiring detailed models to give an insight into the cell rheology. We developed computational model for simulations of cells with nucleus and cytoskeleton in flows in complex domains such as capillary networks and microfluidic devices. We validated the model using experimental data and used it to quantify the effects of cell components on its behavior. We envision that the proposed model will allow to study in silico numerous problems related to the cell biomechanics in flows.

## Introduction

Cell mechanics has proved to be a widely used label-free biomarker to discern phenotypes, detect pathologies and more importantly, monitor existence or progression of a disease [1–3]. The most prominent example is the changes in cell biology and morphology when it evolves from a healthy to a cancerous state [1, 3]. These changes take place at the molecular level affecting properties of individual components of cell internal structure, but eventually leading to alterations in mechanical properties of the whole cell.

Eukaryotic cells are composed of multiple components that contribute diversely to cell mechanics. The most important components are cell membrane, internal cytoskeleton, and nucleus. The cell membrane is a viscous fluid-like matter which consists of various lipids, cholesterol, and embedded proteins. It contributes to cell viscosity, bending resistance, and incompressibility. Cytoskeleton, which is a network of interconnected filaments of different types, connects the cell membrane with underlying sub-cellular components. It is believed to be one of the main contributors to cell mechanics [1]. The nucleus is the largest organelle among sub-cellular components, demonstrating solid-elastic behavior [4], and it is typically stiffer than the cell itself [5]. It is comprised of multiple components including nuclear envelope and chromatin network. Improved understanding of the role that each cell component plays towards cell mechanics may be beneficial for diagnosis and therapy of diseases [2].

One of the novel approaches for studying mechanical properties of cells involves development of custom-designed microfluidic devices where deformability of cells is estimated; this is usually done by measuring the time taken for a cell to pass through a tight straight channel, or its average velocity as it transits through a series of small openings, or by monitoring a cell as it squeezes under hydrodynamic forces [4, 6–9]. These devices can provide higher-throughput systems than conventional technologies such as atomic force microscopy and micropipette aspiration [5] and can be used as a comparative tool between different subpopulations of cells. They, however, often lack in-depth mechanical analysis (ex. elasticity, viscosity) and have little or no regard to the differences in intrinsic properties of these cells.

To obtain a more detailed analysis of the cell mechanics with all its major underlying components, researchers have utilized modeling. Computational approaches to model cell deformation through microfluidic devices as complementary of experimental investigations are prominent for multiple reasons. Firstly, such modeling approaches give an insight into how cell components function under stress. Secondly, they can improve our understanding of the changes that occur during disease progression which, in turn, might uncover reasons for corresponding alterations occurring in cell mechanics [10, 11]. Finally, computational models can be used as predictive tools for the experimental design.

Much progress has been made during the last several years in the field of cell modeling. Mature human red blood cell (RBC) is perhaps among the simplest cells to model, lacking nucleus and internal cytoskeleton. Indeed, membrane models coupled to flow solvers were able to capture essential biomechanical properties of the RBCs in flow. A popular approach is to model the blood plasma with the Lattice-Boltzmann method (LB), RBC membrane forces with finite element method (FE), and RBC-fluid interactions using immersed boundary method (IB) [12–15]. Other models are based on the Finite Volume method [16], moving particle semi-implicit method [17], coarse-grained Molecular Dynamics [18, 19], and Stochastic Rotation Dynamics [20]. RBC models were successfully applied to simulate the flow in capillaries, bifurcations, and microfluidic devices [14, 21–25]. Other cells are composed of, in addition to the cell membrane, a nucleus and internal cytoskeleton. We split the models for cells of this type into two groups. Models in the first group do not explicitly describe nucleus and cytoskeleton, and are based on the RBC membrane model with adjusted parameters set. Examples include recent study that employed such a model to describe cell movement in lateral displacement microfluidic device [26]. The results obtained from the modeling of flow of RBCs, white blood cells and circulating tumour cells (CTCs) were in a good agreement with experiments in microfluidic devices, which primarily rely on separation of cells by size, thus not requiring to reproduce the mechanical properties of cells very accurately in simulations. Some other models are based on the LB-IB method and were applied to CTC membrane deformations [27] and deformable platelet adhesion [28]. The second group of models takes into account sub-cellular components. A platelet model by Zhang *et al.* [29], which consists of 73 thousand particles, was used to simulate dynamic properties of flowing platelets. Ujihara et. al. developed a model to study cell mechanics during tensile test [30]; and Liu et al. studied cytoskeleton deformation during needle injection [31]. The first group of models oversimplifies the cell and does not accurately describe its behavior in situations where the cell undergoes large deformations. At the same time, the computational complexity of the other existing models makes their use in the numerical investigation of cell transport in flow difficult.

In this paper, we present a new model that is suitable for modeling of cells with wide range of viscoelastic properties and, at the same time, computationally efficient to be employed to large and complex flow domains. We developed a robust numerical framework employing Dissipative Particle Dynamics. To the best of our knowledge, this is the first study that models significant flow-induced deformations of cells using mesoscale particle-based model that explicitly takes into account cell membrane, nuclues, and internal cytoskeleton. To validate our model experimentally, we have chosen normal breast epithelial cells (MCF-10A). Using micropipette aspiration experiment, we first probed cells’ elastic properties and used these data to set up parameters of the computational model. We then validated the model using data from microfluidic experiments, where MCF-10A cells were flown through three different microfluidic devices with a series of triangular shaped constrictions and transit velocity of these cells was measured. Using our computational model, we related transit velocity to cell elastic and viscous properties and examined the effect of cell model components such as cytoskeleton and nucleus on whole cell mechanics. We envision that the developed model will bring us closer to understanding the role of cell biomechanics in the broad spectrum of phenomena, such as the mechanical consequences of the structural changes that occur as cell evolves from healthy to diseased state.

## Materials and methods

### Experimental setup and material preparation

#### Cell line

**Fig 1:**
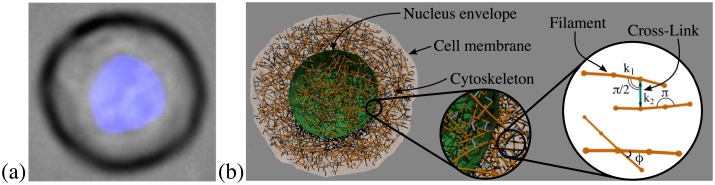
Non-tumorigenic breast epithelial cell (MCF-10A) and its model. a) Microscopy image of a cell with nucleus shown in blue. b) Three component cell model: cell membrane is shown in gray, nucleus is in green, the cytoskeleton is in orange with light blue, the connections between cytoskeleton and membranes are in black. Cytoskeleton network model is composed of long and stiff filaments (orange) connected by short cross-links (light blue).

Human mammary epithelial cell line of MCF-10A was used for the experiment (Fig 1(a)). These cells have composite structure and comprise of several membranes interconnected with numerous filament networks. The plasma membrane is a lipid bilayer which defines the cell border. Mechanics-wise, this membrane is responsible for the cell volume and area conservation and contributes to cell viscosity. It is also connected to the cortical actin layer elastic meshwork. The nucleus is physically integrated with the internal cytoskeleton [32, 33]. It is composed of nuclear envelope and chromatin networks [11]. The nuclear envelope is made of lipid bilayers and underlying thin lamins meshwork which, itself, is binded to the chromatin network [34]. Internal cytoskeleton, the most important contributor to cell stiffness, spans through the cytoplasm from nucleus to plasma membrane. Typically, cells with dense and well-organized cytoskeleton can resist significant stress while those with weaker network are more deformable.

MCF-10A cells were cultured with MEGM (mammary epithelial growth medium) (Lonza, Basel, Switzerland) supplemented with 0.1*μg*/*ml* cholera toxin (Sigma Aldrich, USA) and were maintained at 37°*C* in a 95% air and 5% *CO*_2_ incubator (SANYO, Japan). The cell media was replaced with basal media without Fetal Bovine Serum (Thermo Fisher Scientific, USA), and incubated overnight prior to every experiment to synchronize all cell cycles at *G*_0_. Cell size varied between 7.5*μm* and 22*μm* in diameter. To describe the nucleus size, we used nuclear-cytoplasmic ratio (NC). This was estimated from microscopy as a ratio between visible nucleus area divided by cell area. We found that for MCF-10A cells, the NC ratio is 0.29 ± 0.11. Prior to each experiment, cells were harvested using standard cell passaging protocol (330*g* for 5*min*) and resuspended in 0.1% pluronic solution; they were then kept on ice and immediately used for experiment.

#### Micropipette aspiration experiments

**Fig 2:**
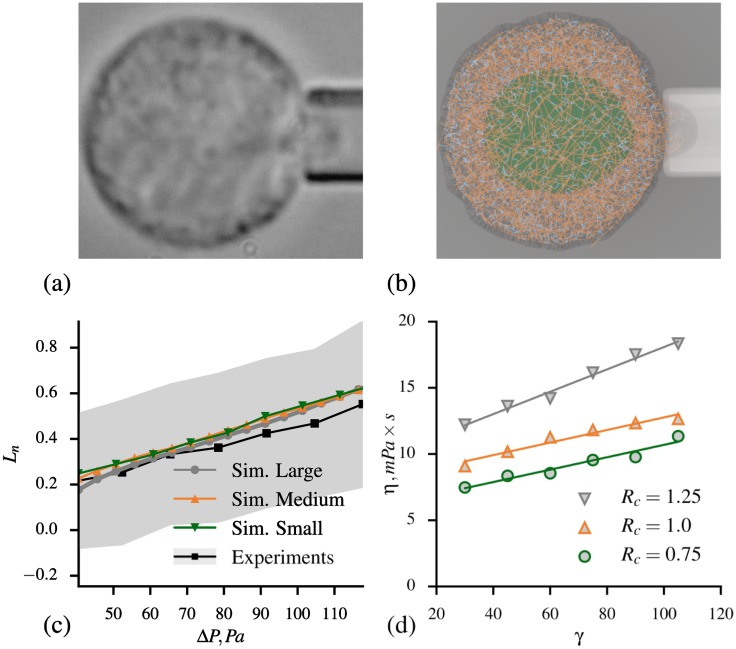
Micropipette aspiration experiments and simulations. a) A representative microscopy of a cell undergoing micropipette aspiration. b) Simulation snapshot of a cell during the micropipette aspiration. c) Comparison between experimental data and simulation for micropipette aspiration, where *L*_*n*_ is normalized indentation length and Δ*P* is aspiration pressure. The gray area represents standard deviation for experimental data, standard deviation bars for simulations are omitted as they are smaller than the symbols. d) Cell viscosity, *η*, as a function of dissipative force parameter *γ* and cutoff length *R*_*c*_ obtained from micropipette aspiration simulations.

We employed micropipette aspiration technique to measure the elastic properties of cells [32, 35]. Prior to each experiment cells were suspended in 10% Bovine Serum Albumin (BSA, Sigma Aldrich) to minimize cell to pipette adhesion. During each pipette aspiration measurement, a cell was placed next to a micropipette of radius *R*_*p*_ and increasing suction pressure was applied, driving portion of the cell into the micropippete (see Fig 2(a)). The suction pressure was increased from 0 to 117.72*Pa* with a constant rate of 3.27*Pa*/*s*. Cell deformation was recorded at 2 frames per second. The cell response during this experiment can be described by a normalized aspiration length $L_{n}=\left(L_{p}-L_{p}^{0}\right)/R_{p}$, where *L*_*p*_ is an aspiration length which depends on the applied pressure and elastic properties of the cell, while constant $L_{p}^{0}$ is aspirated length at tiny pressure. Measurements of normalized aspiration length were used to define parameters of the simulation model of the cell as will be described later in the text. These measurements can further be used to estimate elastic modulus of each cell under the assumption of the cell being a homogeneous elastic solid using Theret et al. model [36]. The estimated elastic modulus of MCF-10A was found to be 237.47 ± 66 Pa.

#### Microfluidic device experiments

**Fig 3:**
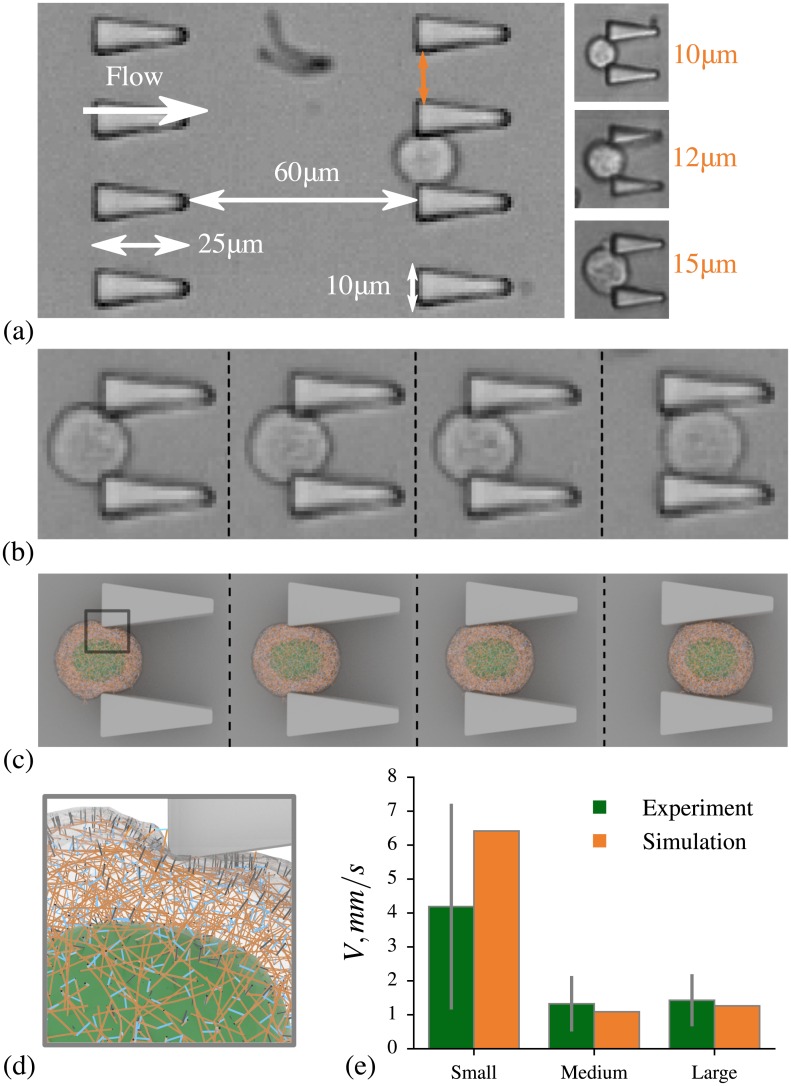
Microfluidic experiments and simulations. a) Microscopy image of section of the device. b) Microscopy of a MCF-10A cell squeezing between obstacles. (c-d) Simulation snapshots for a MCF-10A cell model squeezing between two diverging constrictions. Fluid particles are omitted. e) Comparison between experiments and simulations for the cell velocities, error bars represent standard deviation.

In our experiments, we used three different microfluidic devices with varying constriction gap sizes. For each device, microfluidic master wafer was fabricated using standard soft-lithography techniques. Microfluidic chips were fabricated in polydimethylsiloxane (PDMS) (Sylgard 184 Silicone Elastomer Kit, Dow Croning, USA) by double casting process. The working part of all three devices consisted of ten rows of triangular shaped constrictions as shown in Fig 3(a). The depth of the devices was 25.8*μm*, while the spacing between the rows of obstacles was chosen to be 60*μm* (more than three times the average cell diameter). To compensate for variability in cell size, we have divided cell population into three categories of small (*D* = 10 − 14*μm*), medium (*D* = 14 − 18*μm*), and large (*D* = 18 − 22*μm*) cells, where *D* stands for the cell diameter. In order to study each category, we used microfluidic devices which differ in gap size between the obstacles: device I with 10*μm* gap size for small size cell population, device II with 12*μm* gap size for medium cell population, and device III with 15*μm* gap size for large cell population. Prior to each experiment, microfluidic chips were washed for 10 minutes with 1% BSA to reduce cell-surface friction. Primed chips were placed under a 20x objective of an Olympus IX71 microscope for brightfield imaging. The cells were counted with BIO-RAD TC20 Automated Cell Counter and were diluted with a 0.1% pluronic solution to obtain desired cell concentration of 200*kcells*/*ml* to ensure that devices are not overwhelmed with too many cells. A pressure pump (Eleveflow, France) was used to drive the cells into the constrictions and a high-speed camera (phantom V9.1) was used to record cell passage as avi with 1400 frames per second. Pressure drop was optimized to be 0.67*Pa*/*μm* to provide ample time for cell recovery after each deformation in all devices. As cells were flown into the devices, they had to deform their way through the constrictions and differences in average velocity was determined by these cells’ mechanical properties. The recorded videos were processed using a custom-written ImageJ [37] macro to eliminate the constrictions and to threshold the cells. The thresholded videos were further processed using IMARIS 8.1 tracking algorithm (Bitplane, Switzerland) to track each cell by surface rendering. Transit velocity of each cell was then calculated by dividing its traveled distance over time.

### Cell model

We developed a new computational model mimicking structure of a cell. The model has three components: cell membrane, nuclear envelope, and internal cytoskeleton, as shown in Fig 1(b). The cell membrane model describes lipid bilayer together with underlying cortical actin layer. The nuclear membrane with lamins meshwork are defined by the nucleus envelope model. Both membrane models are described in Section Membrane model. The cytoskeleton is represented by a network of cross-linked filaments mimicking the topology of F-actin network, see Section Cytoskeleton model. For the sake of simplicity, the chromatin network inside the nucleus is described by the same model. The random cytoskeleton network formation and its integration with the membranes are explained in Section Cell model formation. To model fluid and fluid-structure interactions, we used the Dissipative Particle Dynamics method [38–40], described in Section Dissipative Particle Dynamics.

Although we explicitly model sub-cellular components, we follow the phenomenological approach, rather than reductionist method, to explain the properties of a system from the properties of its constituents [10]. This is due to our limited knowledge of the cell mechanics on the considered length scale. The aim of our cell model is to describe the correct mechanics of the whole cell, rather than to accurately reproduce the mechanical response of each individual molecular constituent at the microscale. Thus, although we, where it is possible, incorporate the knowledge of the microscale mechanics, we do not state that the model describing every particular constituent is correct. The important implication of this is that many model parameters can not be directly related to experimentally measured properties of individual cell constituents.

#### Dissipative Particle Dynamics

Dissipative Particle Dynamics (DPD) is a particle-based method which in recent years has been extensively used to study many biophysical systems [40–50]. The popularity of this method is due to several essential properties. First, DPD provides an accurate hydrodynamics due to mass and momentum conservation [51]. It also allows to model complex interactions between particles representing fluid, cell, and solid walls in a unified way by defining DPD interaction parameters. Finally, DPD is a scale-free method meaning that it can be used for modeling processes on different length scales, from nanometers to microns and above. Another consequence of a scale-free property is that DPD does not explicitly imply a unit mapping. Hence, the properties of the fluid or immersed bodies, such as viscosity or elasticity, are determined a posteriori.

Fluids in DPD are modeled by particles with the choice of the number density governed primarily by the computational efficiency [39]. We set the DPD fluid number density to be equal to 3 in our simulations, which is within the typical range of 3 − 4 used in literature. The time evolution of positions and velocities of DPD particles in the system are described by Newton’s equations of motion,

$$\begin{array}{c} \frac{dr_{i}}{dt}=v_{i} \end{array}$$ (1)

and

$$\begin{array}{c} \frac{dv_{i}}{dt}=f_{i}\;, \end{array}$$ (2)

where **r**_*i*_ and **v**_*i*_ are position and velocity of particle *i*, respectively.

The force **f**_*i*_ which acts on particle *i* is expressed by three additive parts,

$$\begin{array}{c} f_{i}=\sum_{j\neq i}\left(f_{ij}^{C}+f_{ij}^{D}+f_{ij}^{R}\right)\;, \end{array}$$ (3)

which are non-zero within a cutoff radius *R*_*c*_. The conservative force $f_{ij}^{C}$ is a soft repulsion force, acting along the vector between particles *i* and *j*, with a parameter *a* defining the maximum repulsion between the two particles,

$$\begin{array}{c} f_{ij}^{C}=\{\begin{array}{cc} a\left(1-r_{ij}/R_{c}\right){\hat{r}}_{\text{ij}}, & r_{ij}<R_{c}\\ 0, & r_{ij}\geq R_{c} \end{array}, \end{array}$$ (4)

where **r**_*ij*_ = **r**_*i*_ − **r**_*j*_, *r*_*ij*_ = |**r**_*ij*_| and ${\hat{r}}_{ij}=r_{ij}/\left|r_{ij}\right|$.

The dissipative force, $f_{ij}^{D}$, and the random force, $f_{ij}^{R}$ are expressed as

$$\begin{array}{c} f_{ij}^{D}=-\gamma w^{D}\left(r_{ij}\right)<{\hat{r}}_{ij},v_{ij}>{\hat{r}}_{ij}\;, \end{array}$$ (5)

and

$$\begin{array}{c} f_{ij}^{R}=\sigma w^{R}\left(r_{ij}\right)\theta_{ij}{\hat{r}}_{ij}\;, \end{array}$$ (6)

where **v**_*ij*_ = **v**_*i*_ − **v**_*j*_, and *θ*_*ij*_ = *θ*_*ji*_ stands for a random variable with zero mean and unit variance, <, > is a dot product. Parameters *a*, *γ*, and *σ* control the strength of conservative, dissipative and random forces, respectively. The last two forces form the DPD thermostat and are related by the fluctuation dissipation theorem as *w*^*D*^(*r*_*ij*_) = [*w*^*R*^(*r*_*ij*_)]^2^ and *σ*^2^ = 2*γk*_*B*_ *T* [51]. We use a generalized weighting function *w*^*R*^(*r*_*ij*_) = (1 − *r*_*ij*_/*R*_*c*_)^0.25^ [52]. Values of conservative and dissipative force parameters used in simulations are summarized in Table 1. For filament-filament and filament-cell interactions, we employed different *R*_*c*_ for conservative force (0.5) and for thermostat (1.0). The short cut-off radius for repulsion together with high value of corresponding conservative force parameter allows to prevent filaments from crossing each other, while it is desirable to have the freedom in varying *R*_*c*_ and *γ* for the thermostat to model cytoskeletons of different viscosities. Cell membranes interactions are strongly repulsive following previous works [53]. For the time integration, we used DPD time step equal to *dt* = 10^−3^. Depending on the system, simulations were run between 5 × 10^5^ and 5 × 10^6^ time steps.

**Table 1:** DPD parameters listed in the format of *a*/*γ*. Values shaded with yellow describe interactions with *R*_*c*_ = 0.5 for repulsive interaction while *R*_*c*_ = 1 for thermostat. For dark gray, *R*_*c*_ = 0.5. The parameters values have been obtained from simulations.

	1. Fluid	2. Filaments	3. Membrane	4. Nucleus	5. Wall
1	10/30	10/45	4/45	4/45	10/30
2		100/65	100/65	4/65	4/65
3			100/45	100/45	10/45
4				100/45	10/45
5					excl.

#### Membrane model

Cell membrane and nucleus envelope are described by the viscoelastic membrane model used previously in RBC simulations [53–58]. This model is briefly reviewed next, whereas detailed description of the model is available elsewhere [54]. The membranes are modeled by a triangular mesh with *N*_*v*_ vertices, where each vertex is represented by a DPD particle. The model takes into account the elastic energy, bending energy, and constraints of fixed surface area and enclosed volume; the total energy is defined as

$$\begin{array}{c} U=U_{s}+U_{b}+U_{a}+U_{v} \end{array}$$ (7)

where *U*_*s*_ is the elastic energy that mimics the elastic meshwork (cortical actin layer for the cell or lamins network for nucleus), given by

$$\begin{array}{c} U_{s}=\sum_{i\in \text{springs}}[\frac{k_{B}Tl_{max}}{4p}\frac{3x_{i}^{2}-2x_{i}^{3}}{1-x_{i}}]\\ +\sum_{\alpha \in \text{triangles}}\frac{C_{1}}{A_{\alpha }} \end{array}$$ (8)

where *k*_*B*_ *T* is the energy unit, *A*_*α*_ is the area of triangle *α* formed by three vertices, *x*_*i*_ = *l*_*i*_/*l*_*max*_, *x*_0_ = *l*_0_/*l*_*max*_, and *l*_*i*_ is the length of spring *i*, *l*_0_ and *l*_*max*_ are the equilibrium spring length and maximum spring extension, *p* is the persistence length, *C*_1_ is constant which depends on *l*_*max*_, *p* and *l*_0_ [59].

The membranes viscosity is imposed by introducing a dissipative and random forces on spring particles, which have the form [60],

$$\begin{array}{c} F_{ij}^{D}=-\gamma^{T}v_{ij}-\gamma^{C}<v_{ij},{\hat{r}}_{ij}>{\hat{r}}_{ij}, \end{array}$$ (9)

$$\begin{array}{c} F_{ij}^{R}dt=\sqrt{2k_{B}T}(\sqrt{2\gamma^{T}}d\bar{W_{ij}^{S}}+\sqrt{3\gamma^{C}-\gamma^{T}}\frac{tr[dW_{ij}]}{3}1)\cdot r_{ij}, \end{array}$$ (10)

where *γ*^*T*^ and *γ*^*C*^ are dissipative parameters (*γ*^*T*^ = 3*γ*^*C*^), **v**_*ij*_ is the relative velocity of spring particles, and $d\bar{W_{ij}^{S}}=dW_{ij}^{S}-tr\left[dW_{ij}^{S}\right]1/3$ is the traceless symmetric part of a the Wiener increments matrix, the elements of which are from *N*_0,1_ distribution.

The bending resistance of the membrane is modeled by

$$\begin{array}{c} U_{b}=\sum_{\alpha ,\beta \;\text{pair}}k_{b}[1-\cos(\theta_{\alpha \beta }-\theta_{0})], \end{array}$$ (11)

where *k*_*b*_ is the bending modulus constant, *θ*_*αβ*_ is the instantaneous angle between two adjacent triangles having a common edge, and *θ*_0_ is the equilibrium angle. The membranes model includes the area and volume conservation constraints, which mimic the area-incompressibility of the lipid bilayer and the incompressibility of the interior fluid, respectively. The corresponding energy terms are given by

$$\begin{array}{c} U_{a}=\frac{k_{a}k_{B}T{\left(A-A_{0}\right)}^{2}}{2l_{0}^{2}A_{0}},\;U_{v}=\frac{k_{v}k_{B}T{\left(V-V_{0}\right)}^{2}}{2l_{0}^{3}V_{0}} \end{array}$$ (12)

where *k*_*a*_ and *k*_*v*_ are the area and volume constraint coefficients. The terms *A*_0_ and *V*_0_ are the equilibrium surface area and enclosed volume of the membrane, respectively.

#### Cytoskeleton model

In order to describe the viscoelastic properties of the internal cytoskeleton, we developed a new mesoscopic model.

The cytoskeleton of a typical cell is composed of multitude of linked molecules with different elastic properties [61]. It is impractical to explicitly model them for the problems on the chosen length scale. Instead, we propose a simplified model which involves filaments and cross-links of only one type, where every filament in our model represents a bunch of filaments rather than individual ones. We emphasize that our model was not built to capture the microscopic details of a real cytoskeleton, but rather it was developed to be relatively easy to parametrize and to have a modest computational complexity.

We took advantage of the recent progress in the cytoskeleton network modeling and employed ideas developed in a series of papers dedicated to F-actin network modeling by Kamm et. al. [62–64]. In the Kamm et. al. model, semi-flexible actin filaments are represented as a series of cylindrical segments, connected by elastic hinges [65]. Cross-linking proteins (CLs) are modeled similarly. The harmonic potentials are employed to describe the extension and bending of both actin filaments and cross-links. Although in this model filaments and CLs are expressed by a linear elastic elements, it was shown that they semi-qualitatively capture experimentally observed behavior characteristic for semi-flexible networks [62]. Other authors, who use similar approaches, also report that the network as a whole appears to have non-linear deformation properties [30, 66]. For the cytoskeleton model used in this work shown in Fig 1(b), we adopted general structure and potentials from Kamm et. al. model. Yet our model is different in several aspects. First, we use 1*μm* as unit of length while for Kamm et. al. model, it is 70*nm*, which leads to a different parametrization. Selected length scale also potentially limits the range of deformations the model can describe accurately. It may be feasible to overcome this limitation in the future by developing a multiscale model where the level of coarse-graining can be chosen arbitrary, similarly to previously developed multiscale RBC model [54]. Second, we employ much smaller unit of time because the aim of the presented model is to study relatively short-term deformation which do not exceed 10*s*. Third, the aim of our cytoskeleton network is to model not only the viscoelastic effect of F-actin proteins, but also the resistance to compression which is believed to be due to different types of cytoskeletal proteins. Finally, the model was implemented within the DPD framework, which allowed us to seamlessly couple entire cell mechanics with surrounding fluid.

Forces acting on particles forming cytoskeleton are classified by the number of bodies involved. Two and three body potential among adjacent filament particles are described by the harmonic law:

$$\begin{array}{c} E_{bond}=\kappa_{fil}{\left(r-r_{0}\right)}^{2},\;E_{angle}=\kappa_{bend}{\left(\theta -\theta_{0}\right)}^{2} \end{array}$$ (13)

where *κ*_*fil*_ is spring constant, *r*_0_ is equilibrium length, *κ*_*bend*_ is bending stiffness, and equilibrium angle *θ*_0_ = *π*. The same potentials are used for CLs with corresponding parameters *κ*_*CL*_, *κ*_*CL*/*fil*_, and *θ*_0_ = *π*/2. The model parameter values are summarized in Table 2. We also use four body interactions to model torsion between two filaments connected by CL, which is described by bending potential Eq (11). Without the additional torsion force we could not achieve required stiffness with the chosen CL and filaments concentration.

**Table 2:** List of major parameters with their values. The source of the parameter values is in the last column. If a value is taken from literature, the reference is given. Parameters, for which values were found through simulation in Sections Cytoskeleton model and Membrane model are marked with ^‡^ and * symbols, respectively.

Parameter	Phys. units value	Sim. units	Source
Filament length	4.0*μm*	4.0	[67]
Filament number density, *N*_*fil*_	-	3.5	^‡^
Filament spring constant, *κ*_*fil*_	0.092*N*/*m*	8 × 10^4^	^‡^
Filament bending stiffness, *κ*_*bend*_^ ‡^	4.025 × 10^−16^ *J*	350	^‡^
Cross-links number density, *N*_*CL*_	-	0.525	^‡^
CL spring constant, *κ*_*CL*_	0.0092*N*/*m*	8 × 10^3^	[69]
CL-filament bending stiffness, *κ*_*CL*/*Fil*_^‡^	6.325 × 10^−16^ *J*	550	^‡^
CL-filament torsion stiffness, *k*_*tor*_	4.7 × 10^−16^ *J*	470	^‡^
Persistence length, *p*	0.00141*μm*	0.00141	[54]
Viscosity parameter, *γ*_*C*_	1.15 × 10^−9^ *Ns*/*m*	30	[68]
Cell area cons. constant, *k*_*A*_	-	10000	[53]
Cell volume cons. constant, *k*_*V*_	-	15000	[53]
Cell spring max length, $l_{max}^{cell}$	3.0*μm*	3	*
Cell bending stiffness	6.14 × 10^−18^ *J*	65	*
Nucleus area cons. constant, *k*_*A*_	-	5000	*
Nucleus volume cons. constant, *k*_*V*_	-	15000	[53]
Nucleus spring max length, $l_{max}^{nucl}$	1.2*μm*	1.2	*
Nucleus bending stiffness	2.6 × 10^−17^ *J*	250	[54]

#### Cell model formation

The random filament network is generated by the procedure mimicking formation of F-actin networks [62]. First, a periodic domain is filled in randomly with filaments of length 4.0*μm* [67]. The density of filament particles is in the range of around 3.5 particles per cubic micron. This value was chosen to provide us with relatively uniform and dense cytoskeleton structure. It also allows us to model cells with wide range of elastic moduli. Second, we simulate spontaneous polymerization and depolymerization of the filaments. During this process, particles from one end of a filament can unbind, while a new particle can be added to another end of a filament. A particle binds if the distance to the closest filament’s end is less than 0.5*μm*. A particle unbinds with the probability proportional to the difference between number of added and removed particles obtained on the previous iteration. Thus, the total length of the filaments remains approximately constant during the simulation. We run this simulation for 10^4^ time steps.

At the next stage, auxiliary cross-link particles with density of 0.525 particles per cubic micron are added to the system. We employ auxiliary CL particles instead of connecting filaments directly because it significantly simplifies the control over the number of connections between filaments. The chosen concentration of cross-links to filaments ratio is within the range used in other works [69]. Formation of cross-links is simulated as follows. If the distance *r* between a CL particle and a filament is less than *r*_0_ = 0.25*μm*, they bind. One CL particle can bind only to two different filaments. One filament particle can have only one CL and this CL cannot bind filament to itself. The CL is able to unbind in a force-dependent manner following Bell’s equation [70]:

$$\begin{array}{c} \kappa \left(F\right)=\{\begin{array}{cc} \kappa^{0}\exp\left(\frac{\lambda F}{k_{B}T}\right) & \;\text{if}\;r\geq r_{0}\\ \kappa^{0} & \;\text{if}\;r<r_{0} \end{array} \end{array}$$ (14)

where *κ*^0^ is a zero-force unbinding rate constant, λ is the mechanical sensitivity for unbinding, and *F* is the magnitude of the force acting on this bond. We set *κ*^0^ = 78*s*^−1^, λ = 3.5 × 10^−5^ *μm* in this work. We utilize this equation to simulate the stochastic nature of bond rupture [64]. This simulation is performed until the number of free CL particles converge to the minimum. Typically, this process takes 6 × 10^5^ time steps.

Once the network has been generated, we post-process it to make it computationally more efficient. We first delete CL particles which are attached to only one filament, since they do not contribute to the cytoskeleton mechanics. We then delete other CL particles, merging two bonds between each CL particle and filaments into one CL bond connecting pair of filaments.

The obtained random filament network is incorporated with the cell membrane and nucleus envelope. First, we cut part of the network which corresponds to the cell volume and simulate spontaneous formation of connections between the membrane vertices and filaments. The new bond is created if the distance between filament and membrane particles is less than 0.5*μm*; for the nucleus, the chosen distance is 0.4*μm*. One filament particle can be connected to only one membrane particle. The unbinding of the newly created bonds happens on force-dependent manner following Eq 14 with parameters $\kappa_{cell}^{0}=30s^{-1}$, λ_*cell*_ = 10^−4^ *μm*, $\kappa_{nucl}^{0}=78s^{-1}$, λ_*nucl*_ = 2 × 10^−4^ *μm* for cell and nucleus correspondingly. This simulation runs until number of connected membrane vertices converges. At the next step, we add four body potential to model resistance of two connected filaments to the torsion with respect to connecting CL. The equilibrium angle for a dihedral is set to the initial value of torsion angle between connected filaments after the cell generation. The resulting cytoskeleton network topology can be characterized by the following properties. The number of CLs per filament is 2.1 ± 1.1, the number of cell membrane particles binded with cytoskeleton relative to the total number of membrane particles is 0.21, similar value for nucleus membrane is 0.26.

We do not consider cytoskeleton reorganization after the cell model has been generated, since the timescale of these processes is too large in comparison with the timescale of deformations in microfluidic devices. Namely, polymerization and depolymerization take up to 10*s* and 100*s* correspondingly [71] and CL unbinding requires at least 2.5*s* [72].

## Results and discussion

We employ two different setups in simulations. We start with calibration and validation of the model for MCF-10A cells of medium size (*D* = 16*μm*). Specifically, we first calibrate the model parameters using micropipette aspiration simulations to match viscous and elastic properties of the cell model with experimental measurements (Section Calibration of cell model, micropipette aspiration). We then validate the model using data obtained by flowing medium size cells through microfluidic device II, see Section Validation of cell model, microfluidic device II. With calibrated model parameters, we create models for small (*D* = 12*μm*) and large (*D* = 20*μm*) cell groups, by changing only the size of the model. We perform micropipette aspiration simulations to measure elastic and viscous properties of two cell models. We then perform further validation using data from experiments with two other microfluidic devices, i.e. device I for small and device III for large size cell populations, see Section Validation of cell model, microfluidic devices I and III. Further, we examine how the structural properties of the cytoskeleton, such as filament and cross-links densities, affect the mechanical behavior of cells based on the proposed model (Section Effect of the cytoskeleton). Finally, in Sections Effect of nucleus and Effect of the cell viscosity, we characterize the impact of nucleus size and cell viscosity on the mechanical response of the cell.

### Calibration of cell model, micropipette aspiration

In this section, we provide the rational behind the choice of model parameters. We consider the model of medium size MCF-10A cell, which has diameter of *D* = 16*μm* with nuclear-cytoplasmic ratio of *NC* = 0.29. We first focus on parameters defining elastic properties of the cell, while parameters affecting cell viscous properties are considered later. We explain the choice of parameters grouped by sub-cellular components.

Cell membrane model describes both lipid bilayer and cortical meshwork. Although some parameters can be directly extracted from experimental data, such as cell and nucleus size, the experimental values of others are unknown. It is widely accepted though that the main contributor to the cell stiffness is the internal cytoskeleton and the impact of the membrane is not that significant. Thus, we model membrane as relatively soft material and we base its parameters on the values previously used for RBC membrane modeling (persistence length, viscosity, bending stiffness). We represent cell surface with triangular mesh with 3500 vertices, so that the average bond equilibrium length is 0.518*μm*. The stiffness of the membrane model can be controlled by the value of the maximum elongation of the WLC link *l*_*max*_. Considering experimentally observed values of elastic modulus for different cell lines, we found that *l*_*max*_ = 3.0*μm* is a reasonable choice. This rather high value allows us to model even very soft cells, increasing the range of model applications.

The cytoskeleton model has more parameters with unknown values than other components. The procedure used to generate the network was explained previously in Section Cytoskeleton model. Parameters of the cytoskeleton model which regulate stiffness of filaments and cross-links, namely spring and angle constants (see Eq 13), have to be specified. Since every filament in the model represents a bunch of protein filaments of different origin, we cannot relate the model parameters describing them with the molecular-level data. Instead, we have chosen such values which result in the correct estimated elastic modulus for the whole cell model. We used relatively stiff filaments with high bending rigidity, while CLs are one order of magnitude softer which is usually the case for actin networks [63]. Variation of filament-CL torsion stiffness can be used as an additional way to control the overall stiffness of the entire cytoskeleton network.

Parameters for the nucleus envelope, which in our model describes both lipid membrane and underlying lamins network, must be defined as well. To minimize the total number of parameters in the cell model, we chose nucleus envelope membrane parameters to be equal to corresponding parameters in the cell membrane model. The only parameter which we vary to control the nucleus stiffness is *l*_*max*_, which was set to 1.2.

To define the values of model parameters mentioned above, we performed a series of simulations of micropipette aspiration experiments (see Fig 2(b)), which allowed us to estimate elastic properties of the whole cell model. Specifically, we tuned model parameters until the desired properties of the cell were obtained. We note here, that the resulting set of parameters is not uniquely defined. It is possible that some of the parameters can be chosen in a more rigorous way. However, since our goal is to match the properties of the entire cell, we are not concerned here with particular values of parameters describing individual constituents of the model. The resulting set of parameters is listed in Table 2. To verify that this set of parameters gives acceptable results, we performed micropipette simulations with 16 independently generated cells with each cell rotated by 4 different angles. For every case, we examined the dependence between applied aspiration pressure *δP* and normalized aspirated length *L*_*n*_. We compared mean *L*_*n*_ among all the simulations for each *δP* with the corresponding mean values obtained from the experiments and found a good agreement, as shown in Fig 2(c).

The estimation of cell viscous properties usually requires a more complicated analysis than needed for elastic modulus estimation. For the current simulation study, cell viscous properties were determined using the time dependence of micropipette aspiration length at constant applied pressure. The longer it takes for a cell to reach plateau value of aspirated length, the higher is its viscosity. We perform this analysis using an extension of the Theret model proposed by Guevorkian et al. [73] which provides a procedure for more accurate viscosity (*η*) estimation than the original model. In order to control *η*, we use cutoff radius *R*_*c*_ and dissipative force coefficient *γ* parameters for interaction between filaments and other particles. We observe that the viscosity positively correlates with both parameters and the highest value of viscosity can be achieved by setting both *R*_*c*_ and *γ* to the highest possible values, see Fig 2(d). The proposed model allows to vary viscosity up to 3 times which might be useful for modeling cells of different types. For MCF-10A, experimentally observed values for *η* are in the range of 6.75 − 13.75*mPa* × *s* [74]. Thus, we chose *R*_*c*_ = 1.0 and *γ* = 65 to have *η* = 10*mPa* × *s*.

### Validation of cell model, microfluidic device II

With the model parameters defined by measuring the whole cell response in micropipette aspiration simulations, we perform validation of the model using experimental data for medium size MCF-10A cells traversal through microfluidic device II described in Section Microfluidic device experiments.

In simulations, the geometry of microfluidic device is described by the signed distance function. The no-slip boundary conditions on the solid walls are implemented using the bounce-back reflection coupled with layers of frozen DPD particles inside the wall [75, 76]. We set up the pressure difference, driving the flow in the working part of microfluidic device in simulations, by matching the average velocity (22.57*mm*/*s*) of small 3.85*μm* beads which we added to the flow in experiments. We considered the same 16 cell models used in the micropipette aspiration simulations and modeled their passage through microfluidic device. In Fig 3(b) we show snapshots of a typical cell squeezing between the obstacles in an experiment. Corresponding snapshots obtained in simulations are shown in Fig 3(c) and 3(d). Comparison of mean cell velocity in simulations and experiments is shown in Fig 3(e). The results are in a good agreement.

### Validation of cell model, microfluidic devices I and III

In the previous section, we validated the cell model using data from microfluidic experiments with medium size cells in device II. In this section, we will consider two other devices, I and III, which were used with small and large size cell populations, respectively. The microfluidic experiments in devices I and III were performed with the same pressure difference driving the flow as in device II.

The difference between microfluidic devices I, II and III is the gap size between the obstacles which was chosen based on the average cell size (see Fig 3(a)). Specifically, the ratio between the gap size and the average cell diameter in device III is the same as in device II and is equal to 0.75. Device I, on the contrary, has the ratio between the gap size and the average cell diameter equal to 0.83. The experimental results (Fig 3(e)) showed that the average velocity is approximately the same in devices II for medium size cells and device III for large cells. The average velocity of small size cells in device I was found to be much higher than for the medium size cells in device II.

We created models for small and large size cells using the same procedure and parameters as used for the medium size cell model. The only difference was the diameters of the cell, which for small and large cell models were 12*μm* and 20*μm*, respectively. We employed 16 independently generated cell models for each cell size. The micropipette aspiration setup was used to estimate elastic properties of all cells. Applying the same procedure as for medium cells, we found dependence of normalized aspiration length *L*_*n*_ on pressure *δP* for small and large cell groups, see Fig 2(c). We do not observe impact of the cell model size on *L*_*n*_ and, thus, on the elastic properties of cells. Viscosity is also the same for all cell models. In Fig 3(e) we show the results from microfluidics simulations performed with small and large size cell models. Similar to experiment, we observe that the average velocity of small size cells in device I is much larger than the velocity of medium and large size cells in devices II and III. In general, simulation results and experimental data are in a good agreement.

Although the interactions of cells with the obstacles in microfluidic devices are complex, some simple considerations may help explain the observed results for three devices. One of the important parameters is the effective size of the opening between the adjacent obstacles in the devices. More specifically, we can define the effective size as a radius of the circle with the same area as the area of the opening. The values which we obtain for three devices then are 9.1, 9.93 and 11.1. Another important parameter, is the ratio between the effective size of the openings and the average cell diameter. The values we obtain for three devices are 0.76, 0.62 and 0.55.

The effective size of the openings in device III is roughly 1.1 times larger than in device II. Therefore, we expect the average fluid velocity to be higher in device III comparing to device II. At the same time, the ratio between the opening size and the cell diameter is smaller in device III, and therefore the cells in device III have to squeeze through the opening which has smaller relative size, making it more difficult to pass. These two effects partially cancel each other, and the resulting average cell velocities are approximately the same in the two devices.

If we consider devices I and II, the effective size of the opening is smaller in device I comparing to device II, so we expect the average fluid velocity to be smaller. At the same time, the ratio between the opening size and cell diameter is larger in device I, making it easier for cells to pass through device I. The last effect strongly dominates the reduction in the average fluid velocity and, hence, the velocity of the small cells in device I is several times higher.

Interactions of cell with the obstacles in all three microfluidic devices make accurate modeling of mechanical properties of cell essential to obtain correct prediction of the average cell velocity. With the help of simulations, we now should be able to analyze and explain such interactions in more details.

### Effect of the cytoskeleton

The cytoskeleton is believed to be one of the main contributors to cell stiffness. During progression of several diseases, changes in cytoskeleton structural properties may lead to significant softening of the cell. Such alternations include reduction of the filaments density as well as decrease in number of cross-links. For instance, it has been shown that to facilitate metastasis, cell undergoes a process called epithelial to mesenchymal transition where its cytoskeleton transforms from well-organized network into fragmented arrangement of filaments [77]. By altering cytoskeletal properties, the present model can accommodate for such processes. In this section we perform simulations to quantify how differences in cell internal structures, such as cytoskeleton and cross-links densities, affect cell mechanical properties.

**Fig 4:**
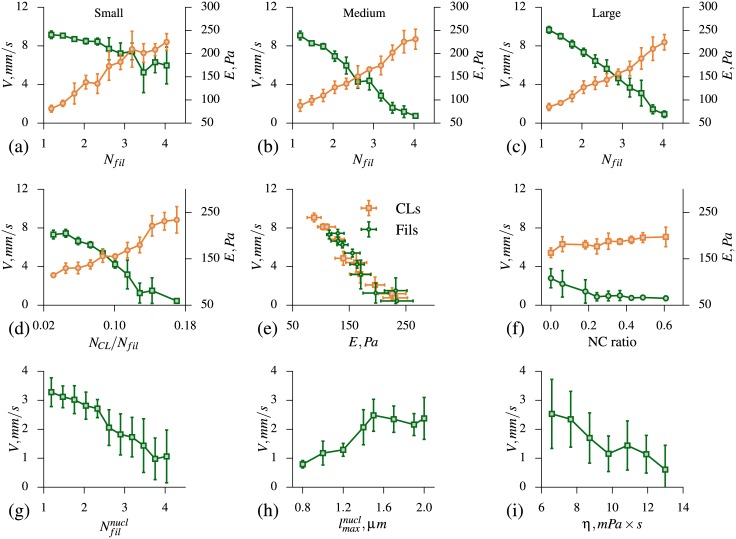
Effects of cytoskeleton, nucleus and viscosity on whole cell model mechanics. (a-c) The simulation results for the effect of the cytoskeleton filaments number density *N*_*fil*_ on elastic modulus (orange) and velocity (green) of small, medium and large size cells. (d) Influence of the cross-links *N*_*CL*_ to filaments *N*_*fil*_ density ratio on elastic modulus (orange) and on velocity (green). (e) Influence of elastic modulus on the velocity for the case when the stiffness is changed by varying filaments density *N*_*fil*_ (green) or cross-links density *N*_*CL*_ (orange). (f) Dependence of cell elastic modulus and velocity on nuclear-cytoplasmic ratio. (g) Effect of filaments number density representing chromatin inside the nucleus on cell velocity. (h) The impact of nuclear laminar properties varied using parameter $l_{max}^{nucl}$ in the nucleus membrane model on the cell velocity. (i) Effect of viscosity on cell velocity in microfluidic device. Error bars on all plots show standard deviation.

To simulate cytoskeleton density variations, we use filaments number density parameter, *N*_*fil*_ (model parameters are listed in Table 2). By varying its value between 1.25 and 4, we obtain elastic modulus between 75*Pa* and 260*Pa* in micropippete aspiration simulations, demonstrating strong dependence of cell stiffness on the cytoskeletal density. This dependence appears to be similar for all cell sizes as shown in Fig 4(a)–4(c). Cytoskeletal density also affects the velocity of cells in microfluidic simulations. As expected, the average cell velocity is lower with higher cytoskeleton density for all cell sizes. However, devices II and III appear to be more sensitive, demonstrating faster decrease of cell velocity, in comparison with device I, see Fig 4(a)–4(c). Smaller ratio of effective opening size to average cell diameter in these devices results in larger relative cell deformations. This suggests that devices II and III are more suitable for studying the effect of cytoskeleton structure variation.

Cross-links density, *N*_*CL*_, is another parameter which significantly affects the cytoskeleton properties. In our model, we can vary this parameter directly by changing the number of CLs particles during cytoskeleton network generation. We examined the impact of *N*_*CL*_ on elastic modulus for medium size cell model and found that this parameter is as significant as cytoskeleton density.*N*_*CL*_ can alter elastic modulus from 110*Pa* to almost 300*Pa*, see Fig 4(d). Dependence of average cell velocity on cross-link density for medium size cells in microfluidic device II obtained in simulations is also shown in the same Figure.

The simulation results allow us to predict dependence of cell velocity in microfluidic device on its elastic modulus as shown in Fig 4(e) for medium size cells in device II. Two sets of results are plotted corresponding to two alternative approaches we used to vary elastic modulus of cells, i.e. by changing the cytoskeleton density or by changing the cross-links density. The agreement between two sets of results provides additional support to one of the assumptions we use in our modeling approach, that not all of the structural constituents at the microscale should be resolved explicitly for the purpose of our studies, as long as whole cell properties are captured accurately.

### Effect of nucleus

The nucleus deformability may be a critical factor in the cells’ ability to pass through small openings. There are two main determinants of nuclear stiffness—nuclear lamina meshwork and the chromatin network inside the nucleus. During the mesenchymal transition, nucleus often becomes bigger and softer. It is known that its softening is primarily due to the chromatin pattern alteration which is the hallmark of malignant nuclei [78]. Altered expression of lamins in a variety of human tumors is also often associated with malignant phenotypes, whether lamins level is upregulated or downregulated depends on the cancer type [79]. Despite current advances in live cell imaging and other biophysical techniques, it is still challenging to study the effect of each component on cells mechanics. In this section, we perform a computational study of the effect of morphological and structural changes of the nucleus. We focus on the medium size cells in microfluidic device II.

First, we vary the size of the nucleus to evaluate its influence on cell elastic modulus as well as its velocity in microfluidic device. We varied NC ratio between 0.0 (no nucleus) and 0.7 for medium cells with elastic modulus of around 180*Pa*, see Fig 4(f). We have chosen a cell with relatively low elastic modulus because cells with very large nucleus tend to get stuck in microfluidic device. We observed that the nucleus size has a minor impact on results of micropipette aspiration simulations, indicating maximum increase of elastic modulus, *E*, only by 17% comparing to the cell model without nucleus. Results from microfluidics simulations, on the contrary, suggest that nucleus plays an important role in cell passage as shown in Fig 4(f). In particular, in the absence of nucleus (*NC* = 0.0), we observed approximately 2.5 times increase in average velocity in comparison to cells with nucleus of normal size (*NC* = 0.29). From the modeling prospective, it means that it is essential to explicitly model nucleus for the considered type of cells.

To study the effect of chromatin concentration, we vary the filaments number density inside the nucleus ($N_{fil}^{nucl}$) while keeping the density of the cytoskeleton filaments constant. Our results suggest that the chromatin network has a significant impact on the cell stiffness, see Fig 4(g). For example, if the network is very sparse ($N_{fil}^{nucl}=1.25$), the velocity increases significantly, exceeding the velocity of the cell model without nucleus. We note here, that for *NC* = 0.0, the cell interior is completely filled by cytoskeleton with filament density *N*_*fil*_.

Next, we study the effect of the nuclear lamina on cell traversal in microfluidic device. The density of the nuclear lamina meshwork is modeled in the present study by parameter $l_{max}^{nucl}$ in the nucleus membrane model. By varying $l_{max}^{nucl}$, we observe that by reducing stiffness of the envelope, we can again increase the average cell velocity significantly, see Fig 4(h).

Our simulation results indicate that the impact of the nucleus on cell traversal through microfluidic device cannot be explained primarily by nucleus size, nuclear lamina or chromatin networks contributions, but rather all components may significantly alter cell dynamics.

### Effect of the cell viscosity

Cell viscosity is yet another property that may affect cell passage through microfluidic device. In general, dependence of cell velocity in microfluidic device on its viscosity can be non-trivial. In our previous studies with healthy and malaria infected RBCs in microfluidic device of similar design [7], the device was found to be sensitive mostly to elastic properties of cells. Due to the specific interplay between the time needed for a cell to travel from one row of obstacles to the next and RBC characteristic relaxation time, the average cell velocity was almost independent of its viscosity. The distance between rows of obstacles and the driving pressure gradient were set in experiments so that RBCs did no have enough time to completely recover their shape during passage from one row of obstacles to the next. Cells with higher viscosity required longer time to deform and squeeze between pair of obstacles. However, these cells also required longer time to recover their shape, and therefore approached the next pair of obstacles with the shape making passage through the opening easier.

In the devices used in the present study, cells recover their shape almost completely. Therefore, we do not expect similar effects to take place. Indeed, the results of simulations with medium size cells in microfluidic device II show roughly linear decrease of average cell velocity with increasing viscosity, as one would expect (Fig 4(i)). The cell viscosity was varied in simulations by changing cutoff radius *R*_*c*_ and dissipative force coefficient *γ* parameters for interaction between filaments and other particles. The obtained dependence shows that viscous properties of the cell can have comparable effect on its traversal to cell elastic properties.

### Conclusion

We developed a new eukaryotic cell model which takes into account cell membrane, cytoskeleton and nucleus. The non-tumorigenic breast epithelial cells (MCF-10A) were used in our studies. To estimate the viscoelastic properties of cells and to calibrate our computational model, we performed micropipette aspiration experiments. The model was then validated using data from three microfluidic experiments with devices designed to take into account size variation in MCF-10A cell population. We note here, that the chosen set of model parameters may not be unique and better agreement particularly for small cells in microfluidic device I may be achieved given that there are many parameters in the proposed model. However, we want to emphasize that we did not use any data from microfluidic experiments to set cell model parameters. Taking into account the interplay of average flow velocity and cell interactions with obstacles in microfluidic devices, the fact that the model can predict (even not perfectly but still within the experimental error bars) cell velocities is quite remarkable in our opinion. Additional validation and benchmark tests are necessary to tune the model more carefully.

Using the model, we probed contributions of sub-cellular components to whole cell mechanics in micropipette aspiration and microfluidics experiments. We obtained that the main contributor to cell stiffness is its cytoskeleton. This finding is in agreement with previous experimental studies [80–82]. Our model showed that both filament and cross-links concentrations play equally important role in defining whole cell mechanics, dominating over the effects due to variation of cell nucleus properties. Simulation results indicate that it is important to model nucleus explicitly in microfluidics simulations. Each of considered nucleus properties, namely nucleus size, stiffness of nuclear lamina and chromatin network, can significantly affect deformability of the cell. The viscous properties of the cell can have comparable effect to cell elastic properties on its traversal through microfluidic device.

We believe that the new model will allow to study in silico numerous problems in the context of cell biomechanics in flows in complex domains, such as capillary networks and microfluidic devices. Our ongoing work indicates that the proposed cell model parametrization has the flexibility to be used in simulations of various cell types, including cancer cells with different mechanical properties. With further development, the present model with explicit description of sub-cellular components may be used to study different alterations in cell mechanics caused by diseases or functional changes.
